# Towards a Sustainable Material Protection: Olanzapine Drugs and Their Derivatives as Corrosion Inhibitors for C1018 Steel in 1 M Hydrochloric Acid

**DOI:** 10.3390/ma18122902

**Published:** 2025-06-19

**Authors:** Habibah M. A. Omar, Nestor Ankah, Mohamed S. Gomaa, Malak Y. Alkhaldi, Nadir M. A. Osman, Abdullah R. Al-Subaie, Ibrahim Aldossary, Irshad Baig, Ashraf A. Bahraq, Marwah Aljohani, Ihsan Ulhaq Toor, Aeshah H. Alamri

**Affiliations:** 1 Chemistry Department, College of Science, Imam Abdulrahman Bin Faisal University, P.O. Box 76971, Dammam 31441, Saudi Arabia; 2230000255@iau.edu.sa (H.M.A.O.); mhaljohani@iau.edu.sa (M.A.); 2 Interdisciplinary Research Center for Advanced Materials (IRC-AM), King Fahd University of Petroleum & Minerals (KFUPM), Dhahran 31261, Saudi Arabia; nestor.ankah@kfupm.edu.sa; 3 Department of Pharmaceutical Chemistry, College of Clinical Pharmacy, Imam Abdulrahman Bin Faisal University, P.O. Box 1982, Dammam 31441, Saudi Arabia; msmmansour@iau.edu.sa; 4 Department of Chemistry, King Fahd University of Petroleum and Minerals, Dhahran 31261, Saudi Arabia; malak.alkhaldi@kfupm.edu.sa (M.Y.A.); nadirosman@kfupm.edu.sa (N.M.A.O.);; 5 SPIMACO Group, Operations, Dammam Pharma Plant, Plant Technical, Dammam 31441, Saudi Arabia; ibrahim.aldossary@spimaco.sa; 6 Basic and Applied Scientific Research Center, Imam Abdulrahman Bin Faisal University, P.O. Box 1982, Dammam 31441, Saudi Arabia; ibaig@iau.edu.sa; 7 Interdisciplinary Research Center for Construction and Building Materials, King Fahd University of Petroleum & Minerals, Dhahran 31261, Saudi Arabia; ashraf.bahraq@kfupm.edu.sa; 8 Department of Mechanical Engineering, King Fahd University of Petroleum and Minerals (KFUPM), Dhahran 31261, Saudi Arabia

**Keywords:** corrosion inhibition, carbon steel, pharmaceutical compounds, olanzapine derivatives, DFT, molecular dynamics

## Abstract

This study investigates the synthesis process and characterization methods and evaluates the inhibition behavior of olanzapine (2-methyl-4-(4-methyl-1-piperazinyl)-10H-thieno-[2,3-b] 1,5]benzodiazepine (OLZ)) and its derivatives, such as 3-(2-methyl-4-(4-methylpiperazin-1-yl)-10H-benzo[b]thieno[2,3-e] [1,4]diazepin-10-yl) propenamide (OLZ1) and Ethyl 2-(2-methyl-4-(4-methylpiperazin-1-yl)-10H-benzo[b]thieno[2,3-e][1,4]diazepin-10 yl) acetate (OLZ2) for carbon steel (C1018) in a 1 M HCl acidic solution. Fourier Transform Infrared Spectroscopy (FTIR) and Nuclear Magnetic Resonance (NMR) were employed to verify their molecular structures and functional groups, which characterized the derivatives after synthesis. Their corrosion inhibition potential for C1018 steel in acidic media was estimated by weight loss (WL) and electrochemical techniques, such as electrochemical impedance spectroscopy (EIS), linear polarization resistance (LPR), and potentiodynamic polarization (PDP), accompanied by surface analysis methods. The findings revealed that all three derivatives demonstrated exceptional inhibition performance, achieving maximum efficiencies of 88.83%, 91.20%, and 91.82% for OLZ, OLZ1, and OLZ2 at 300 ppm, respectively. Weight loss experiments across different temperatures further explored their inhibitory behavior. Although inhibition efficiency decreased with a temperature increase to 318 K, the derivatives still displayed notable performance, with maximum efficiencies of 74.75% for OLZ, 81.63% for OLZ1, and 79.44% for OLZ2. Polarization studies identified the corrosion inhibition mechanisms as an anodic type. Surface characterization of the C1018 steel coupons, both with and without the inhibitors, was performed using FTIR and scanning electron microscopy (SEM) combined with energy-dispersive X-ray spectroscopy (EDX). These analyses indicated the creation of a protective inhibitor layer on the carbon steel surface, reducing corrosion in the acidic environment. Overall, this study underscores the potential of these drug derivatives as corrosion inhibitors, combining structural insights and performance assessments to support their industrial application.

## 1. Introduction

Corrosion has been identified as a major challenge in industry due to the harsh environmental conditions that prevail in Saudi Arabia. Acidic corrosion is the most common in industrial fields, such as oil and gas, chemical processing, and metal manufacturing. In 2022, for example, it accounted for 32.4% of Saudi Arabia’s GDP. However, corrosion remains a costly challenge, with global losses estimated at USD 2.5 trillion annually, or 3.4% of the world’s GDP, according to NACE International [1]. In the U.S. oil and gas industry alone, corrosion costs approximately USD 1.4 billion annually in exploration and production, with additional expenses of USD 7 billion for monitoring, maintenance, and replacement in gas and liquid transmission pipelines [1,2].

Developing effective corrosion inhibitors is fundamental to minimizing these major economic losses. Organic compounds with heteroatoms such as oxygen, nitrogen, and sulfur, together with π-electron systems, represent the main components of conventional corrosion inhibitors, which improve their adsorption onto metal surfaces to boost protective performance [3,4]. However, the environmental toxicity associated with many traditional inhibitors has necessitated a paradigm shift toward sustainable alternatives that maintain high inhibition efficiency while minimizing ecological impact.

Recent research has directed attention toward the potential repurposing of pharmaceutical substances as corrosion inhibitors, particularly those containing N-heterocyclic structures. These compounds demonstrate strong potential to replace traditional inhibitors because they can easily decompose in the environment while showing reduced toxicity and cost-effectiveness [5].

The inherent molecular architecture of pharmaceuticals contains multiple heteroatoms, aromatic rings, and functional groups that allow these substances to create protective films on metals through adsorption reactions and metal ion complexation [6,7]. This structural versatility allows them to provide comprehensive protection against various corrosion manifestations, including uniform, pitting, and crevice corrosion [7]. Several studies have reported the use of drug compounds as corrosion inhibitors containing heteroatoms for inhibiting metal corrosion in an acidic medium (Table 1).

Olanzapine, a typical antipsychotic medication widely prescribed for the treatment of schizophrenia and bipolar disorder, represents a promising candidate for repurposing as a corrosion inhibitor. As a selective antagonist of 5HT2A/D2 receptors, olanzapine has received FDA authorization for multiple psychiatric applications and is listed among the World Health Organization’s essential medicines [8]. The number of annual U.S. prescriptions for olanzapine exceeds 3 million, which positions the medication among the most prescribed drugs [9,10]. The two-year shelf life of olanzapine results in large amounts of expired medication that must be disposed of while also creating opportunities to find sustainable uses for the waste.

**Table 1:** Comparison of different N-heterocyclic drugs that are used as corrosion inhibitors with the results from our study.

No.	Drug Name	Metal/Alloy	Corrosive Medium	Used Methods	Max IE%	Ref.
1	Helicure drug	Carbon Steel	1 M HCl	WL + EIS + EFM	85.8% (at 300 ppm)	[11]
2	Spironolactone drug	n C38 Carbon Steel	10% HCl	WL + EIS + PDP	95.8% (at 7.2 × 10^−3^ M)	[12]
3	Atenolol	Carbon Steel	1 M HCl	WL + EIS + PDP	93.8% (at 300 ppm)	[13]
4	Irbesartan	Carbon Steel	1 M HCl	OCP + EIS + LPR + PDP + CV	94% at 300 mg L^−1^	[14]
5	Metoprolol	Steel alloy (st 37)	1 M HCl	EIS + PDP	87% (300 ppm)	[15]
6	Hydralazine	Carbon Steel	1 M HCl	WL + EIS + PDP	91.44% (1250 ppm at 323 K)	[16]
7	Ciprofloxacin	Carbon Steel	1 M HCl	WL + EIS	69.99% (at 0.7% *v*/*v*%)	[17]
8	Doxofylline	Soft Steel	1 M HCl	WL + EIS	72.6% (200 ppm)	[18]
9	Irnocam	Carbon Steel	1 M HCl	WL + EIS	75.4% (at 600 ppm)	[19]
10	Lumerax	Carbon Steel	1 M HCl	EIS + PDP	97.6% (100 mg/L)	[20]
11	Meloxicam	Carbon Steel	1 M HCl	EIS + PDP	80.8% (30 ppm)	[21]
12	Ornidazole	Carbon Steel	1 M HCl	WL + EIS + PDP	71.61% (2 × 10^−5^ M)	[22]
13	Tramadol	Carbon Steel	1 M HCl	EIS + PDP	97.2% (100 ppm)	[23]
14	Dulcolax	Carbon Steel	1 M HCl	EIS + PDP + AAS	92.5% (500 ppm)	[24]
15	Gentamicin	Carbon Steel	1 M HCl	WL + EIS + PDP	75.06% (0.9% *v*/*v*)	[25]
16	Norfloxacin	Carbon Steel	1 M HCl	WL + EIS + PDP	89.8%	[26]
17	Olanzapine	Carbon Steel	1 M HCl	LPR	92.47	Our work

The molecular structure of olanzapine features multiple nitrogen atoms within heterocyclic rings, aromatic systems with delocalized π-electrons, and functional groups capable of interacting with metal surfaces. These characteristics align with established criteria for effective corrosion inhibitors.

Furthermore, the possibility of synthesizing derivatives with enhanced inhibition properties through strategic molecular modifications offers additional avenues for optimizing performance while maintaining environmental compatibility. The convergence of increasing industrial demand for corrosion inhibitors, growing environmental concerns regarding conventional inhibitors, and the global imperative for sustainable resource utilization underscores the significance of investigating pharmaceutical compounds as potential corrosion inhibitors. This research direction not only addresses critical industrial challenges but also contributes to circular economic principles by transforming pharmaceutical waste into valuable industrial resources.

The present study aims to comprehensively investigate the corrosion inhibition efficacy of olanzapine and two novel synthesized derivatives on C1018 carbon steel in 1 M HCl solution. The research methodology encompasses advanced electrochemical techniques, immersion tests, computational calculations, and surface characterization using FTIR spectroscopy and SEM/EDX analysis to elucidate the inhibition mechanisms and quantify protective performance. By establishing structure–activity relationships and optimizing inhibition parameters, this work seeks to develop environmentally benign corrosion inhibitors derived from pharmaceutical compounds, thereby addressing both industrial corrosion challenges and environmental sustainability imperatives.

## 2. Experimental Section

### 2.1. Synthesis of the Corrosion Inhibitors

**Figure 1:**
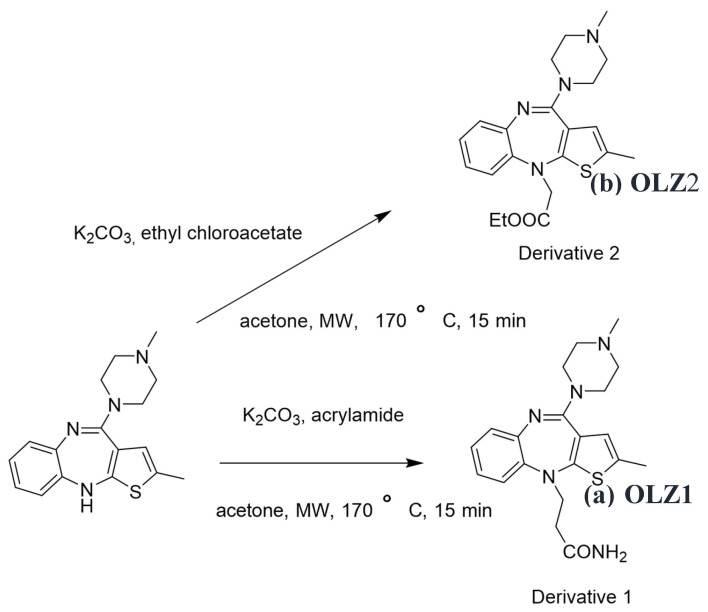
Synthesis of derivatives (**a**) OLZ1 and (**b**) OLZ2.

D-1 (OLZ 1) and D-2 (OLZ 2) were synthesized in neat conditions and under microwave irradiation (Figure 1). The compounds were synthesized through the reaction of olanzapine with acrylamide and ethyl chloroacetate to give D-1 and D-2, respectively, and were recrystallized from acetone. Microwave irradiations were conducted in an Anton Paar Monowave 300 single-mode microwave in 30 mL Pyrex [27,28]. The progress of the reactions was monitored using Merck silica gel 60F-254 thin-layer plates. The compounds were characterized using 1H NMR and FTIR. The 1HNMR spectra were recorded at 500 MHz (Bruker) and IR spectra were recorded on an FTIR (02) spectrophotometer (PerkinElmer, Bruker). The 1HNMR and IR spectra are presented in the Supplementary Materials (Figures S1 and S2) [29].

### 2.2. Weight Loss Measurements

The weight loss or immersion test is one of the primary tests used to estimate the inhibition efficiency of a substance. The sample was prepared, weighed, and then immersed in 250 mL of the solution in a jar with a lid, in the absence and presence of the optimum concentration for each inhibitor (300 ppm), for 24 h. After that, C1018 coupons were taken out and treated by (pickling) to remove oxide contaminants formed on the surface in atmospheric oxygen when the metal is exposed to air [30]. After that, the corrosion rate was calculated [31,32] as follows:

(1) $$Corrosion\;Rate\left(\frac{mm}{y}\right)=\frac{87,600W}{ATD}$$

(2) $$Corrosion\;Rate\left(mpy\right)=\frac{3.45\times 10^{6}\times w}{ATD}$$

(3) $$Inhibition\;Efficiency=\frac{CR{}^{\circ}-{CR}^{i}}{CR{}^{\circ}}\times 100$$

where ***w*** is the coupon weight loss in grams, A is the coupon surface area in cm^2^, T is the immersion period in 24 h, D is the coupon density in g/cm^3^, CR° is the corrosion rate of the solution without an inhibitor, and CR^i^ is the corrosion rate of the solution with an inhibitor.

### 2.3. Electrochemical Measurements

The electrochemical evaluation was carried out using a standard three-electrode corrosion cell consisting of carbon steel (C1018) as the working electrode, silver/silver chloride electrode (Ag/AgCl) as the reference electrode, and graphite rod as the counter electrode. The electrodes were immersed in 300 mL of 1 M HCl solution. Gamry reference 3000 potentiostat/galvanostat/ZRA (Gamry Instrument, USA) was used to perform all the electrochemical measurements. Before testing, the working electrode was immersed in the test solution for 1 h to achieve a stable open circuit potential (OCP). All electrochemical experiments were performed in static, aerated solutions at room temperature. Electrochemical impedance spectroscopy (EIS), linear polarization resistance (LPR), and potentiodynamic polarization (PDP) tests were conducted to assess the corrosion behavior of carbon steel with and without inhibitors. EIS measurements were conducted over a frequency range of 100 kHz to 10 mHz with an AC amplitude of ±10 mV versus OCP. This was followed by LPR tests using a potential scan of ±10 mV relative to the OCP at a rate of 0.125 mV/s. Lastly, PDP tests were carried out by scanning the system at 0.5 mV/s from −250 mV in the cathodic direction to 250 mV in the anodic direction relative to the OCP. The Gamry Echem Analyst 7.9.0 software package was used for data analysis, fitting, and simulations. To evaluate the corrosion rate and inhibition efficiency, all experiments were performed three times to achieve high precision and reliability of the results.

### 2.4. Computational Methods

#### 2.4.1. DFT Calculations

The DFT calculations for the four olanzapine molecules were performed using the Dmol3 module, *Biovia Material Studio* [33]. The GAA/BLYP functional, combined with the DNP basis sets to expand the Kohn−Sham orbitals with a confining cutoff set at 3.8 Å, was employed in the calculations. The COSMO method was applied to simulate the solvent effect of water [34]. The convergence tolerance was set to fine. Based on the frontier orbital theory, the electronic parameters in terms of the highest occupied molecular orbital (E_HOMO_), energy of the lowest unoccupied molecular orbital (E_LUMO_), energy gap (ΔE), electron affinity (A), ionization potential (I), electronegativity (χ), global hardness (η), and the fraction of electron transfer (ΔN) were calculated according to Equations (4)–(9):

(4) $$\Delta E=E_{LUMO}-E_{HOMO}$$

(5) $$I=-E_{HOMO}$$

(6) $$A=-E_{LUMO}$$

(7) $$\chi =-\frac{\left(E_{HOMO}+E_{LUMO}\right)}{2}=\frac{I+A}{2}$$

(8) $$\eta =-\frac{\left(E_{HOMO}-E_{LUMO}\right)}{2}=\frac{I-A}{2}$$

(9) $$\Delta N=\frac{\chi_{Fe}-\chi_{In}}{\eta_{Fe}-\eta_{In}}$$

where χ_Fe_ and η_Fe_ represent the absolute electronegativity and hardness of iron, which have the theoretical values of 0 and 7 eV/mol, respectively [35].

The geometry optimization process was conducted with the following criteria: the convergence tolerance of the energy, maximum force, and maximum displacement were set at 1 × 10^−5^ Ha, 2 × 10^−3^ Ha Å^−1^, and 5 × 10^−3^ Å, respectively, while the SCF convergence tolerance was set at 1 × 10^−6^ and the real space cutoff radius at 4.0 Å.

#### 2.4.2. MD Simulations

A molecular dynamics (MD) simulation was conducted to obtain the interaction energy between the Fe surface and the inhibitor molecules. The Fe(110) crystallographic surface was selected for its stability and abundance of active sites [36]. A supercell of the surface scaled to 10 × 10 times its original size was created and optimized. The interaction models comprised three layers: Fe(110), a solvent with the molecule, and a 30 Å vacuum. The solvent layer was designed to simulate 1 M HCl and included 491 H_2_O, 9 Cl^−^, 9 H_3_O^+^, and 1 inhibitor molecule. All MD simulations were performed using the Forcite module and the COMPASS force field. The models underwent dynamic runs under the NVT ensemble at 298 K, controlled by the Andersen thermostat. The simulation time was 500 ps with a 1 fs time step. The interaction energy was then calculated based on Equation (10):

(10) $$E_{interaction}=E_{total}-\left(E_{surface+solution}+E_{inhibitor}\right)$$

where $E_{total}$ is the total energy of the entire system; $E_{surface+solution}$ is the energy of the surface with the solution; and $E_{inhibitor}$ is the energy of the inhibitor molecule only. The negative magnitude of interaction energy gives the values of binding energy ($E_{binding}$), as expressed in Equation (11).

(11) $$E_{binding}=-E_{inteaction}$$

Several simulation models were constructed to study the diffusion of corrosive particles across the inhibitor films. These models include a blank model containing 180 H_2_O, 5 Cl^−^, and 5 H_3_O^+^, as well as inhibited models with 10 molecules. The built models were optimized using the COMPASS forcefield. Subsequently, dynamics runs were performed (i.e., equilibrium and production runs) under the NVT ensemble at room temperature (298 K). The conformations generated from the MD simulations were used to track the corrosive particles (H_2_O, Cl^−^, and H_3_O^+^) using the mean square displacement (MSD), as expressed in Equation (12). The diffusion coefficients (*D*) of the species were then calculated by fitting the MSD–time curves according to Einstein’s law of diffusion (Equation (13)) [37,38]:

(12) $${∆r}^{2}\left(t\right)=\langle {\left|r_{i}\left(t\right)-r_{i}\left(0\right)\right|}^{2}\rangle$$

(13) $$D=\frac{1}{6N}\underset{t\to \infty }{\lim}\frac{d}{dt}\sum_{i=1}^{N}{∆r}^{2}\left(t\right)$$

where ${∆r}^{2}\left(t\right)$ is the MSD at time *t*; N represents the total number of particles; and $r_{i}\left(t\right)$ and $r_{i}\left(0\right)$ denote the position of a particle i at time t and at the initial time, respectively. The periodic boundary conditions were applied to all surface–molecule interaction models. The Ewald accuracy, cutoff distance, and spline width were set at 1 × 10^−4^ Kcal mol^−1^, 15.5 Å, and 1 Å, respectively. Prior to the production run, an equilibration run was executed at 200 ps NPT.

### 2.5. Surface Analyses

#### 2.5.1. FTIR Analysis

FTIR is a spectroscopic technique that can provide critical information on the interactions between inhibitor molecules and the substrate by detecting changes in the vibrational frequencies of chemical bonds. This information helps us understand the corrosion inhibition mechanism [39]. A small amount of the powder sample was placed directly onto the ATR crystal, without the need for KBr. Gentle pressures were applied to ensure optimal contact between the sample and the crystal. The Shimadzu—IRAFFINITY-2 instrument was used for the FTIR analysis.

#### 2.5.2. Scanning Electron Microscopy (SEM)

The morphology of the C1018 carbon steel surface exposed to uninhibited and inhibited 1 M hydrochloric acid was analyzed using scanning electron microscopy coupled with an energy-dispersive X-ray analyzer (VEGA 3 TESCAN coupled with AMETEK EDX) operated at 20 kV with a 10 mm working distance. SEM can provide information on the morphology and the type of corrosion on the metal surface, while EDX is used for elemental analysis [40].

## 3. Results and Discussion

### 3.1. Weight Loss Measurements

**Figure 2:**
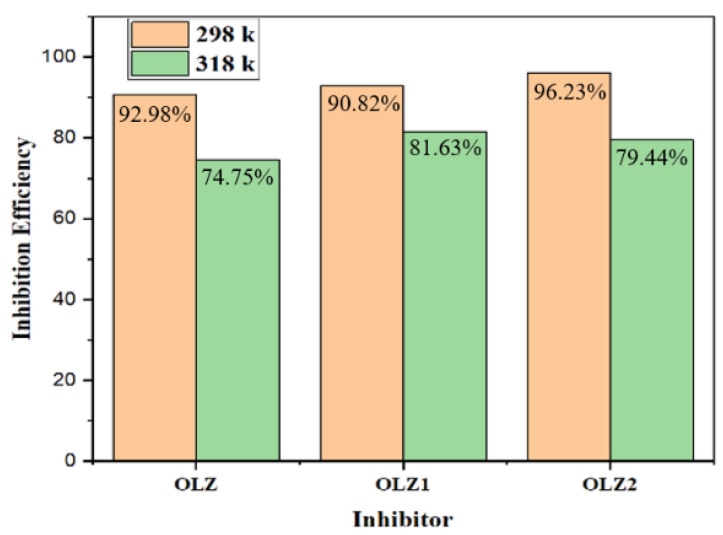
Variation in inhibition efficiency values by weight loss of carbon steel in the absence and presence of inhibitors in 1 M HCl at 298 K and 318 K.

Weight loss measurement was conducted to evaluate the performance of the corrosion inhibitors at 298 K and 318 K. Table 2 and Figure 2 present the inhibition efficiency values for OLZ, OLZ1, and OLZ2 at these two temperatures. At 298 K, the inhibition efficiencies were 92.98%, 90.82%, and 96.23% for OLZ, OLZ1, and OLZ2, respectively, indicating a stronger interaction of the inhibitor molecules at lower temperatures [41,42]. However, at the elevated temperature of 318 K, the inhibition efficiencies decreased to 74.75%, 81.63%, and 79.44% for OLZ, OLZ1, and OLZ2, respectively. The decrease in inhibition efficiency with increasing temperature suggests the desorption of the inhibitor molecules from the metal surface. Interestingly, OLZ1 exhibited the smallest decrease in inhibition efficiency with temperature, suggesting that the acrylamide group enhances the thermal stability of the adsorbed inhibitor film. This could be attributed to stronger interactions between the acrylamide group and the metal surface, possibly through coordination bonds involving the amide nitrogen and oxygen atoms.

### 3.2. Open Circuit Potential Measurements

**Figure 3:**
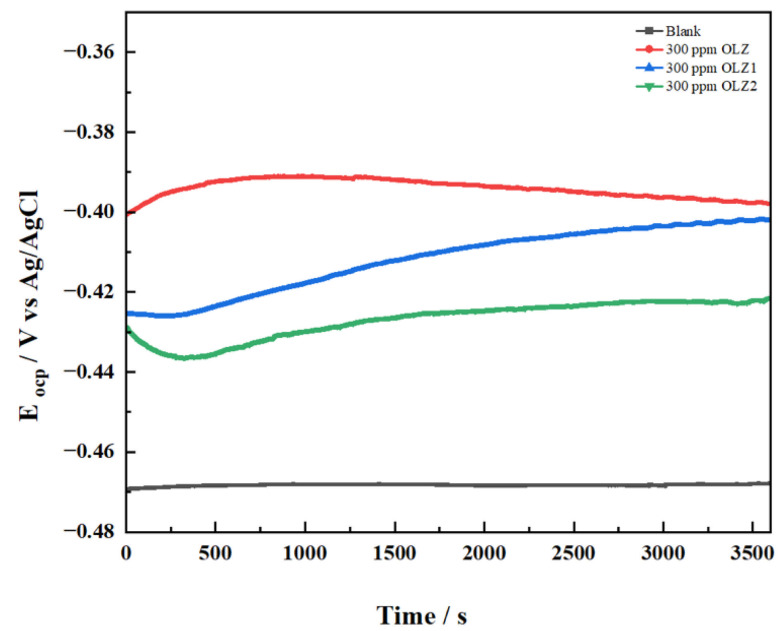
Open circuit potential plots of OLZ, OLZ1, and OLZ2.

Figure 3 presents the OCP versus time plots for the blank solution and solutions containing 300 ppm of OLZ, OLZ1, and OLZ2. In the absence of inhibitors (blank), the OCP stabilized at approximately −0.47 V vs. Ag/AgCl, indicating active corrosion of the carbon steel substrate. Upon the addition of the inhibitors, a notable shift toward more positive (less negative) potential was observed. The OCP values stabilized at approximately −0.40 V, −0.42 V, and −0.44 V for OLZ, OLZ1, and OLZ2, respectively. This positive shift in OCP suggests that all three compounds predominantly affect the anodic reaction of the corrosion process [43]. The OCP measurements also revealed that potential stabilization occurred within the first 1000 s of immersion for all systems, after which minimal fluctuations were observed for the remainder of the 3500 s test period. This indicates the rapid formation of a protective film on the metal surface in the presence of inhibitors [44].

### 3.3. Electrochemical Impedance Spectroscopy (EIS)

**Figure 4:**
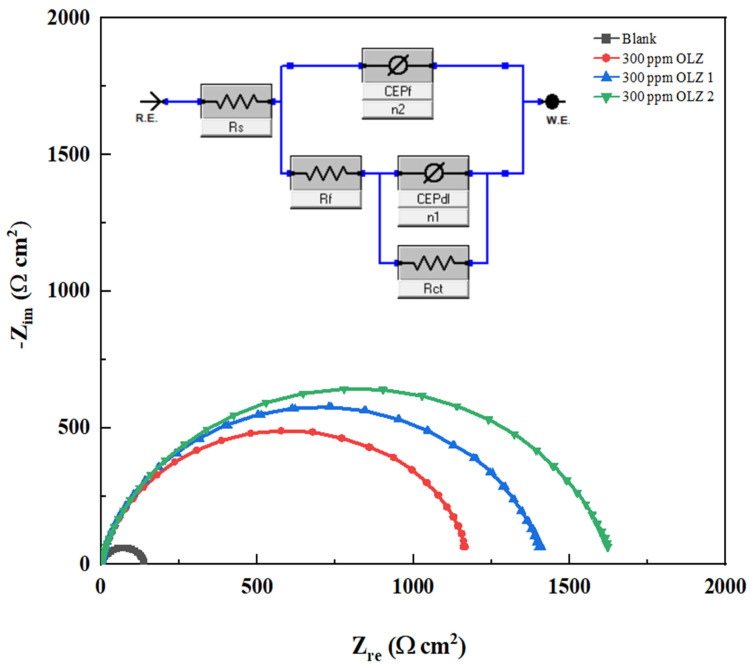
Nyquist plots for the corrosion control of carbon steel in 1 M HCl in the presence and absence of OLZ, OLZ1, and OLZ2.

EIS was employed to analyze the corrosion inhibition on C1018 in 1 M HCl solution, both with and without olanzapine and derivatives (OLZ, OLZ1, and OLZ2). This method reveals both the electrochemical reaction rates and the surface properties of the examined systems. Nyquist plots reveal the corrosion of steel in 1 M HCl solution that consists of semicircles with their centers below the real axis [16], as shown in Figure 4. These characteristics of charge transfer-controlled dissolution with significantly increased diameters in the presence of inhibitors indicate enhanced interfacial charge transfer resistance at the metal/electrolyte interface.

**Figure 5:**
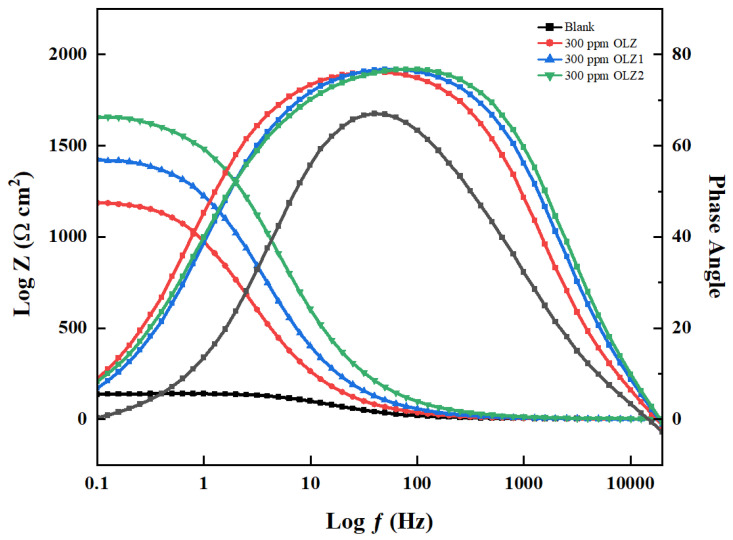
Bode plots for the corrosion control of carbon steel in 1 M HCl in the presence and absence of OLZ, OLZ1, and OLZ2.

The Nyquist plots for the blank and OLZ, OLZ1, and OLZ2 inhibited solutions presented four semicircular capacitive arcs between high- and low-frequency regions; the high-frequency time constant (R_f_//CPE_f_) represented a defective/porous protective OLZ and its derivatives film on the metal surface while the low-frequency time constant (R_ct_//CPE_dl_) represented the double layer formation at the metal/electrolyte interface [45,46]. The Bode plots (Figure 5) further support these findings, showing increased impedance magnitude |Z| and phase angle shifts toward more negative values in the presence of inhibitors. The phase angle plots exhibit a single time constant, which broadens in the presence of inhibitors, indicating the formation of a more compact and protective film on the metal surface [47,48].

The equivalent circuit model used to fit the EIS data is presented in Figure 4 (insert). The parameters include R_s_, which is the solution resistance, CPE_dl_, which is the constant phase element for the double-layer, and ***R*_ct_**, which is the charge transfer resistance. The n values (ranging from 0.867 to 0.996) are less than unity, confirming the non-ideal capacitive behavior of the metal–solution interface. Moreover, higher n values indicate greater surface heterogeneity because of inhibitor adsorption. The observed inductive effects in the EIS data are related to the complex structure of the double layer at the metal–solution interface [49,50]. It is a key indicator of the interface’s electrical properties and inhibitor adsorption characteristics. These ***C*_dl_** values were calculated from the impedance data by applying Equation (14) in Table 3.

(14) $$C_{\mathrm{dl}}\;=\;{(Y}_{º}R_{ct}^{1-n}{)}^{1/n}$$

The *C***_dl_** is the double-layer capacitance, *R***_ct_** presents charge transfer, and *Y***_0_** is the CPE coefficient (reciprocal of impedance and also known as admittance).

The reduction in the electrical double layer value from 193.850 (blank) μF·cm^−2^ to (79.578, 65.471, 51.416) μF·cm^−2^ in the presence of OLZ and its derivatives (OLZ, OLZ1, and OLZ2) indicates an increase in the thickness of the electrical double layer, which retards the corrosion process as presented in Table 3. Also, this decrease in (***C*_dl_**) at the metal–solution interface with the presence of OLZ, OLZ1, and OLZ2 can result from a lower local dielectric constant, which leads to inhibitors adsorbed on the C1018 carbon steel. Quantitative analysis through non-linear least squares fitting to an equivalent electrical circuit revealed a huge increase in faradaic polarization resistance (***R_p_***), from 140.897 Ω·cm^2^ for the uninhibited system to 1200.97, 1430.42, and 1646.42 Ω·cm^2^ for OLZ, OLZ1, and OLZ2, respectively, as shown in Table 3. Inhibition efficiency from the EIS technique was calculated utilizing the relationship defined in Equation (15). The experimental results consistently demonstrate that all three compounds exhibit excellent corrosion inhibition properties, with inhibition efficiencies exceeding 88% at 300 ppm. OLZ2 has the highest inhibition efficiency. This trend suggests that the structural modifications introduced in OLZ1 and OLZ2 enhance the corrosion inhibition properties of the parent molecule.

(15) $$IE\%=\frac{R_{p(inh)}-R_{p(blank)}}{R_{p(inh)}}\times 100$$

***IE*** is the corrosion efficiency, and ***R_p_*** is the polarization resistance. The polarization resistance values without any inhibitor present are denoted by ***R_p_ _(blank)_***, while the values with an inhibitor present are denoted by ***R_p_*_(*inh*)_**.

The superior performance of OLZ2 is attributed to its ethyl acetate functionality, which provides additional oxygen donor atoms for coordinating covalent bonding with d-orbitals of iron and enhanced molecular hydrophobicity. The acrylamide group in OLZ1 showed intermediate performance because of its additional electron-rich centers and π-electron interactions with the metal substrate. The consistent inhibition hierarchy across multiple electrochemical techniques validates the reliability of these findings. This study created structure–activity relationships for olanzapine-based corrosion inhibitors and the results show their effectiveness as protective agents for carbon steel in aggressive acidic environments.

### 3.4. Potentiodynamic Polarization (PDP) Studies

**Figure 6:**
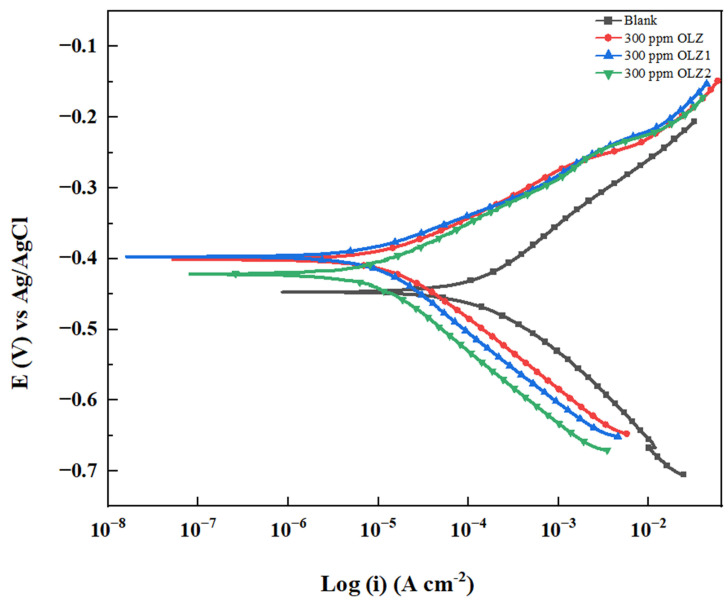
Potentiodynamic polarization plot for the corrosion control of carbon steel in 1 M HCl by different concentrations of inhibitors: OLZ, OLZ1, and OLZ2.

PDP measurements were conducted to further elucidate the corrosion inhibition mechanism of the studied compounds. The polarization curves for carbon steel in 1 M HCl with and without 300 ppm of OLZ, OLZ1, and OLZ2 are shown in Figure 6. The electrochemical parameters of corrosion potential (E_corr_), corrosion current density (*i_corr_*), and inhibition efficiency (% *IE*) values were determined from the corresponding Tafel plots and the obtained data are shown in Table 4. The inhibition efficiencies can be calculated from the *i_corr_* values using the following Equation (16):

(16) $$IE\%=\frac{i_{corr(blank)}-i_{corr(inh)}}{i_{corr(blank)}}\times 100$$

where *i_corr_*_(*blank*)_ and *i_corr_*_(*inh*)_ are the sample’s corrosion current density in 1 M HCl without and with inhibitors, respectively.

The corrosion current density (*i_corr_*) decreased significantly from 200.00 μA cm^−2^ for the blank solution to 20.100, 15.900, and 14.600 μA cm^−2^ for OLZ, OLZ1, and OLZ2, respectively. This reduction in *i_corr_* corresponds to inhibition efficiencies of 89.95%, 92.05%, and 92.70% for OLZ, OLZ1, and OLZ2, respectively, which is in agreement with the EIS results. The corrosion potential (E_corr_) shifted from −447.00 mV for the blank solution to −401.000, −397.00, and −422.000 mV for OLZ, OLZ1, and OLZ2, respectively. All three compounds act as anodic-type inhibitors, affecting the anodic metal dissolution.

The anodic (βa) and cathodic (βc) Tafel slopes were also modified in the presence of inhibitors. The anodic Tafel slope decreased from 160.80 mV/dec for the blank to 100.00, 89.000, and 91.400 mV/dec for OLZ, OLZ1, and OLZ2, respectively. Similarly, the cathodic Tafel slope increased from 120.00 mV/dec for the blank to 132.700, 218.600, and 241.600 mV/dec for OLZ, OLZ1, and OLZ2, respectively. These changes in Tafel slopes indicate that the inhibitors modify the mechanism of both anodic dissolution and cathodic hydrogen evolution, with a more pronounced effect on the anodic reaction. This anodic type of inhibition behavior is further confirmed by the potentiodynamic polarization results, which show that both the anodic branches of the polarization curves are affected by the presence of inhibitors. The changes in both the anodic (***β_a_***) and cathodic (***β_c_***) Tafel slopes indicate that the inhibitors modify the mechanism of both electrode reactions, resulting in a more pronounced increase in anodic Tafel slopes.

### 3.5. Linear Polarization Resistance (LPR)

A linear polarization resistance technique was employed to evaluate the efficiency of OLZ and its derivatives in inhibiting corrosion at 1 M HCl. This measurement enables real-time nondestructive corrosion rate evaluation as an electrochemical assessment tool that researchers commonly use to measure material corrosion rates. The ±10 mV is relative to OCP, which produces a direct relationship between corrosion current and potential values. A small polarization selection maintained an accurate measurement of corrosion rates because it avoided permanent disruption of the corrosion process [51,52].

Table 4 presents both polarization resistance (***R_p_***) and efficiency values. The LPR parameters derived from the curve fitting appear in Table 4. The E_corr_ values of inhibited solutions show more positive values than the blank acid solution, while the E_corr_ values do not follow any specific pattern when using different inhibitors. The inhibition efficiency (% IE) of the OLZ inhibitors was calculated using the following Equation (18) [53]:

(17) $$R_{p}=\frac{\beta_{c}\times \beta_{a}}{2.3\left(\beta_{c}+\beta_{a}\right)i_{corr}}$$

(18) $$IE\%=\frac{R_{p(inh)}-R_{p(blank)}}{R_{p(inh)}}\times 100$$

where ***R_p_*** is the polarization resistance. The polarization resistance values without any inhibitor present are denoted by ***R_p_* _(*blank*)_**, while the values with an inhibitor present are denoted by ***R_p_* _(*inh*)_** and ***i_corr_***, which is the corrosion current density. ***β_a_*** is the anodic and βc cathodic Tafel slope.

The results in Table 4 demonstrate that polarization resistance values increase after adding inhibitors compared to the acid solution without inhibitors. The addition of OLZ together with its derivatives proves effective at blocking corrosion in this extremely acidic environment, as the addition of OLZ and its derivatives produced identical patterns of decreased corrosion rates together with enhanced inhibition efficiencies. The polarization resistance (***R_p_***) increased from 132.20 Ω for the blank solution to 1135.66 Ω, 1473.00 Ω, and 1760.66 Ω for OLZ, OLZ1, and OLZ2, respectively. The corresponding inhibition efficiencies are 88.35%, 91.01%, and 92.47% for OLZ, OLZ1, and OLZ2, respectively, which are in excellent agreement with the values obtained from EIS and PDP.

### 3.6. Quantum Chemistry Calculations

**Figure 7:**
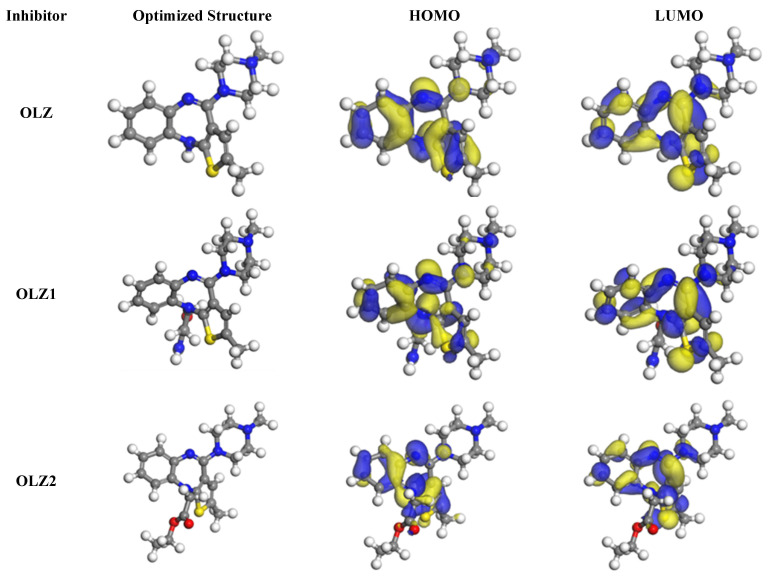
Optimized structures and orbital distributions of the olanzapine derivative molecules.

**Figure 8:**
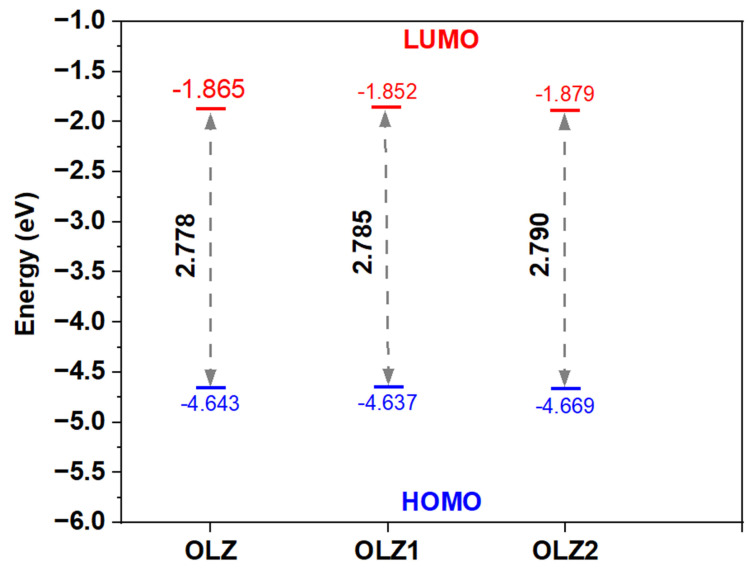
EHOMO, ELUMO, and the energy gap of olanzapine derivative molecules.

This section discusses the results of the quantum chemistry-based calculations. The molecular orbitals play a crucial role in determining the electron transfer process of an organic molecule [54,55]. The frontier molecular orbitals of the olanzapine derivatives are shown in Figure 7 and Figure 8. The HOMOs and LUMOs are concentrated on the OLZ-based molecules, except for the piperazine rings. These locations likely denote and accept electrons on the iron surface during the adsorption process [56]. The reactivity parameters of OLZ and its derivatives are presented in Table 5. The energy gap (ΔE) values of the HOMO-LUMO spectrum for OLZ, OLZ1, and OLZ2 were 2.778, 2.785, and 2.790 eV, respectively. A smaller gap indicates higher chemical reactivity, suggesting good inhibition efficiency [57]. In this study, all molecules showed almost similar *ΔE* values. These theoretical results matched well with the experimental findings based on the electrochemical and weight loss measurements. The dipole moment (*μ*) is another vital parameter in determining a molecule’s effectiveness as a corrosion inhibitor, as it reflects bond polarity. The molecules studied exhibited dipole moments higher than water (1.88 Debye) [58], indicating their potential to outcompete water molecules and adsorb onto the substrate at the metal–solution interface.

**Figure 9:**
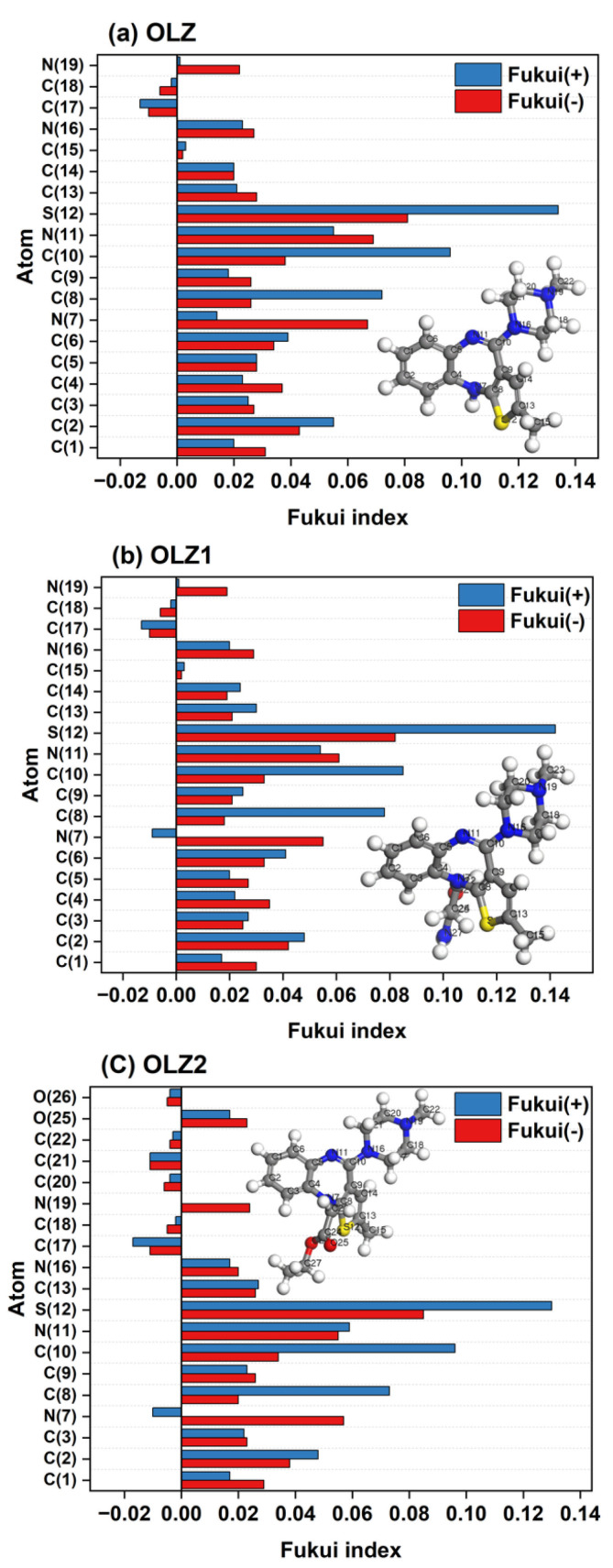
Fukui parameters of the olanzapine-based molecules.

Fukui function based on the Mulliken analysis was performed to characterize the local reactivity of the molecules [59]. The results of the analysis display the *f_k_^−^* and *f_k_^+^* indices of all molecules. The highest *f_k_^−^* values are localized around the nitrogen and sulfur atoms, suggesting these sites are more prone to electrophilic attack. The *f_k_^+^* values are significant in the sulfur atoms in the thiazepine ring, indicating these sites are more prone to nucleophilic attack. These heteroatoms are likely involved in the adsorption process on the steel surface, facilitating the formation of a protective layer as presented in Figure 9.

### 3.7. Adsorption Behavior

**Figure 10:**
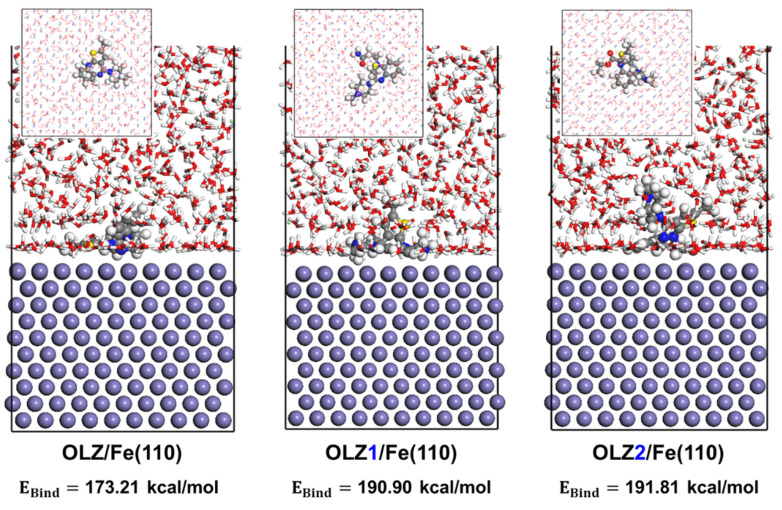
Adsorption configurations of the olanzapine derivative molecules on the Fe(110) surface and their corresponding binding energy.

The interaction of the molecular surface system was investigated using MD simulations to show the adsorption configurations of the inhibitor molecules on the Fe(110) surface in the presence of 1 M HCl, and the results are presented in Figure 10. The adsorption models revealed that the olanzapine derivative molecules were adsorbed on the substrate in a nearly parallel orientation. Although the overall geometry of olanzapine and its derivatives is non-planar due to their three-dimensional structures, their large size allows for more adsorption sites on the metal substrate. This is seen with the OLZ2 molecule, which achieved a more optimal adsorption mode than the others. The adsorption position is crucial for maximizing surface coverage and thereby enhancing the inhibition ability of the molecules [60,61]. Based on the generated dynamic models, the binding energy of all the molecules was calculated, with the results providing values of 173.21, 190.90, and 191.81 kcal/mol for OLZ-Fe(110), OLZ1-Fe(110), and OLZ2-Fe(110), respectively. OLZ2 exhibited the highest binding energy due to its adsorption configuration. These results are consistent with the DFT calculations as well as the experimental findings, which showed that OLZ2 demonstrated excellent inhibition performance in all aspects.

**Figure 11:**
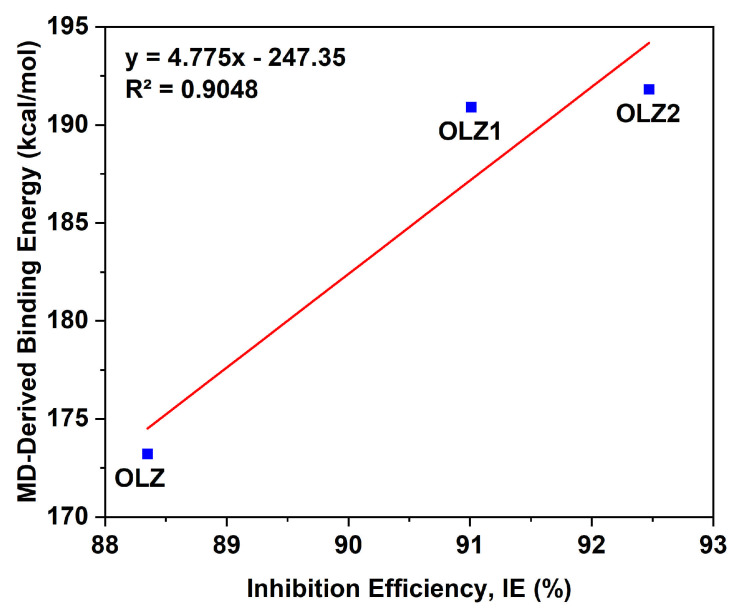
Correlation between MD-derived binding energy and experimental inhibition efficiency for OLZ, OLZ1, and OLZ2.

Figure 11 shows a linear correlation between the IE% and the binding energy derived from the MD simulations. Despite the limited dataset, the trend indicated that molecules with higher binding energies exhibited better inhibition performance. This is consistent with the concept that stronger adsorption of the inhibitor on the steel surface enhances protective behavior. The determination coefficient (R^2^ = 0.9048) confirmed a strong linear relationship, which reinforced the relevance of binding energy as a predictive descriptor in structure–activity relationships. The validity of this correlation is further supported by the fact that MD simulations account for both the inhibitor and solvent interactions, thereby making the correlation more representative of the experimental system.

### 3.8. Diffusion Study

**Figure 12:**
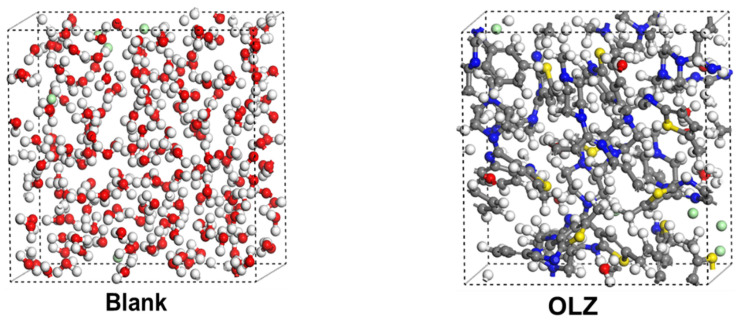

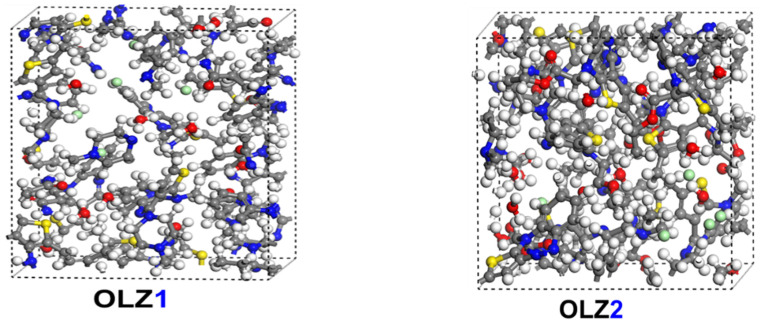
Diffusion models of the blank and inhibited systems.

**Figure 13:**
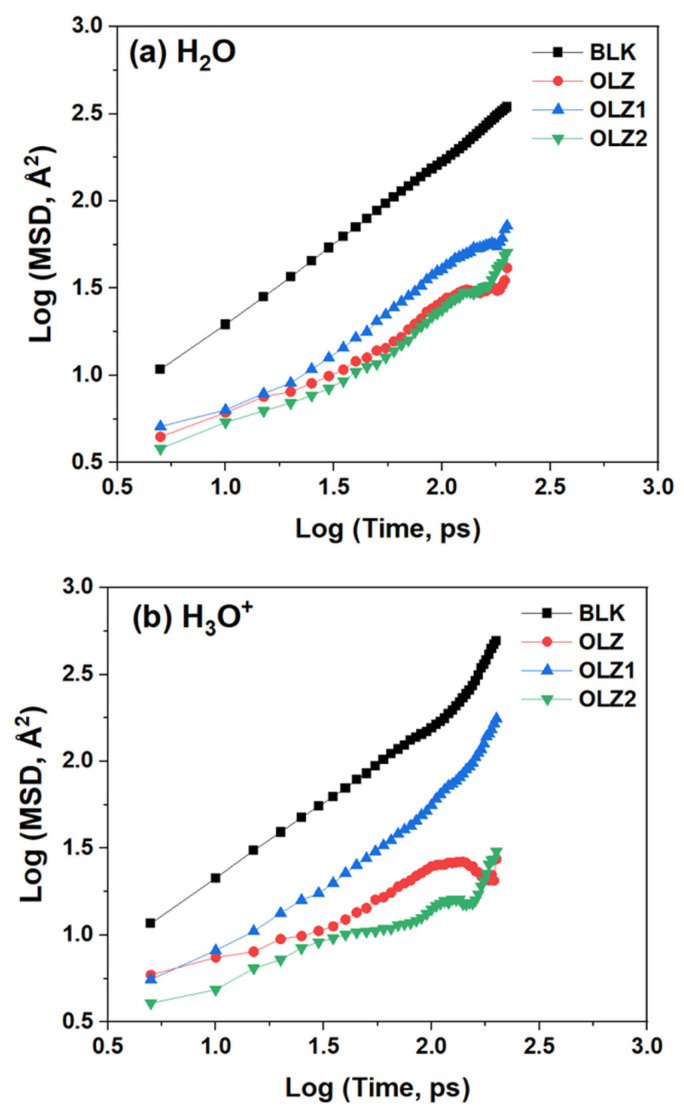

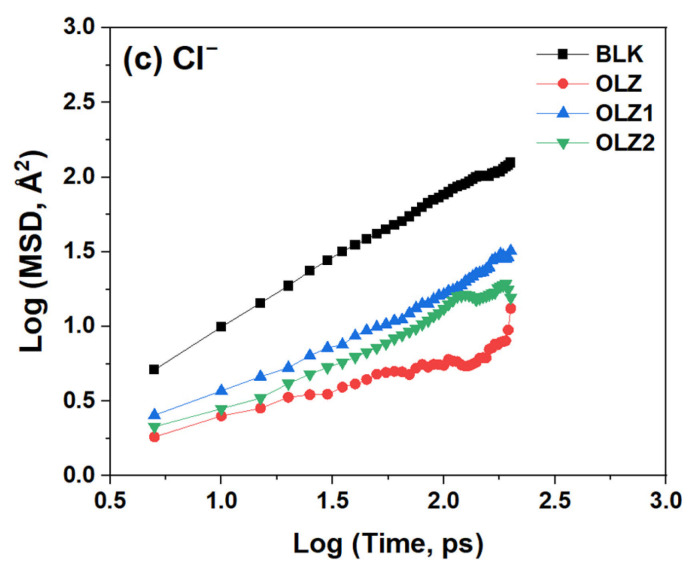
MSD curves of the corrosive particles across the blank and inhibited films: (**a**) H_2_O; (**b**) H_3_O^+^; and (**c**) Cl^−^.

The diffusion models of the blank and inhibited systems are shown in Figure 12 and Figure 13 and Table 6. In principle, the inhibition film should be able to prevent the migration of the species to the metal surface [62,63]. Thus, the MSD analysis was used to track the mobility of the particles in the different inhibitors’ layers, showing the double-log plots of the MSD–time curves for corrosive particles across the blank and inhibited films. The data indicated an increasing trend of MSD values over time, implying the diffusion region of the particles. In addition, the MSD curves for the inhibited systems were significantly lower than those for the blank systems, indicating that the inhibitor molecules restricted particle movement. Based on the MSD data, the diffusion coefficients of H_2_O, H_3_O^+^, and Cl^−^ were calculated, as presented in Table 6. The inhibitor molecules significantly decreased the D-values of H_2_O, H_3_O^+^, and Cl^−^ by around 85%, 83%, and 84%, respectively, on average. It was found that OLZ2 is most effective in reducing the diffusion of H_2_O (0.35 × 10^−9^ m^2^·s^−1^), while OLZ2 and OLZ were more effective for H_3_O^+^ (0.17 × 10^−9^ m^2^·s^−1^) and Cl^−^ (0.07 × 10^−9^ m^2^·s^−1^), respectively.

### 3.9. Surface Analysis

#### 3.9.1. SEM-EDX Analysis

**Figure 14:**
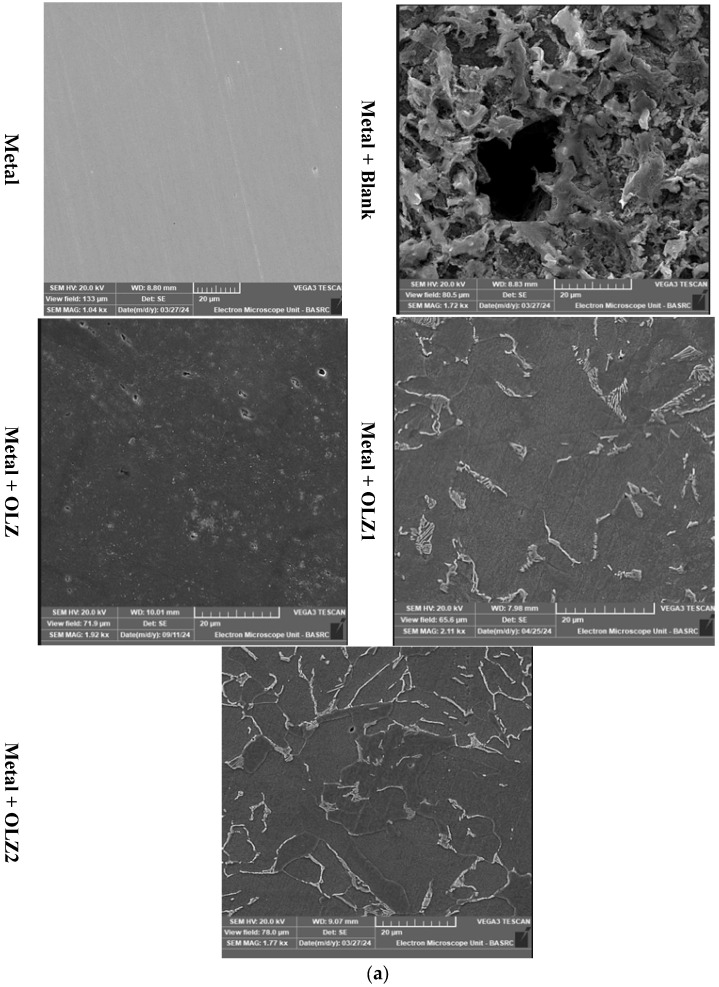

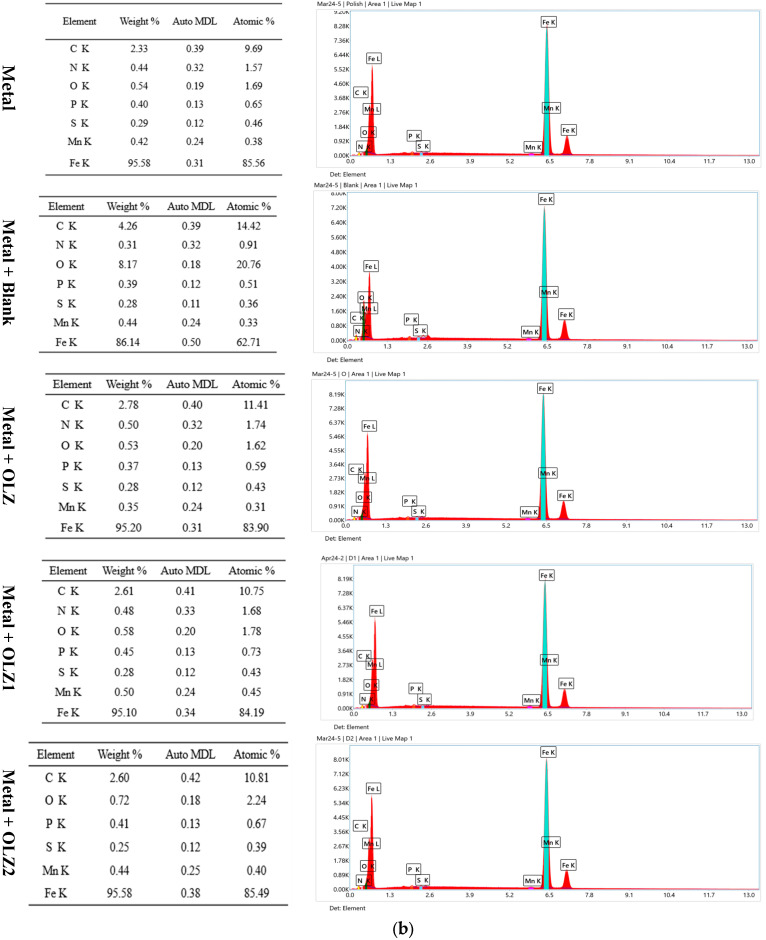
(**a**) Scanning electron micrographs (first row) of C1018 steel samples: polished fresh metal, immersed in 1 M HCl solution and immersed in 1 M HCl solution containing OLZ, OLZ1, and OLZ2. (**b**) EDX graphs (second row) of C1018 steel samples: polished fresh metal, immersed in 1 M HCl so-lution and immersed in 1 M HCl solution containing OLZ, OLZ1, and OLZ2.

Scanning electron microscopy (SEM) coupled with energy-dispersive X-ray spectroscopy (EDX) was used to examine the surface morphology and elemental composition of the carbon steel samples before and after immersion in the test solutions [64,65]. The SEM images and EDX spectra are presented in Figure 14a,b. The SEM image of the bare metal surface shows a relatively smooth morphology with some polishing marks. After immersion in 1 M HCl (blank solution), the surface appears severely corroded with numerous pits and cracks, indicating an aggressive attack by the acidic medium. In contrast, the surfaces of samples immersed in inhibitor-containing solutions exhibit significantly less damage, with smoother morphologies suggesting the formation of protective films. The surface protection appears to be most effective with OLZ2, followed by OLZ1 and OLZ. The EDX analysis presented in Table 7 provides quantitative information on the elemental composition of the sample surfaces. The bare metal surface primarily consists of iron (Fe, 85.56%) with small amounts of carbon (C, 9.69%), oxygen (O, 1.69%), and other elements. After immersion in 1 M HCl, the iron content decreases dramatically to 62.71%, while the oxygen content increases to 20.76%, indicating severe oxidation of the metal surface. In the presence of inhibitors, the iron content is largely preserved (83.90%, 84.19%, and 85.49% for OLZ, OLZ1, and OLZ2, respectively), suggesting effective protection against corrosion. Furthermore, the detection of nitrogen (N) on the inhibited surfaces (1.74%, 1.68%, and 1.68% for OLZ, OLZ1, and OLZ2, respectively) provides direct evidence for the adsorption of inhibitor molecules, as nitrogen is a key element in the molecular structure of these compounds. The carbon content is also higher on the inhibited surfaces (11.41%, 10.75%, and 10.81% for OLZ, OLZ1, and OLZ2, respectively) compared to the bare metal (9.69%), further confirming the presence of organic inhibitor molecules. The oxygen content is significantly lower on the inhibited surfaces (1.62%, 1.78%, and 2.24% for OLZ, OLZ1, and OLZ2, respectively) compared to the corroded surface in HCl (20.76%), indicating reduced oxidation of the metal in the presence of inhibitors.

The surface analysis techniques of SEM-EDX provide direct evidence for the adsorption of inhibitor molecules on the metal surface and the formation of protective films. The SEM images clearly show the protective effect of the inhibitors, with significantly less surface damage compared to the blank solution. The smoother morphology of the inhibited surfaces suggests the formation of a uniform protective film that shields the metal from the aggressive medium. The EDX analysis confirms the presence of inhibitor molecules on the metal surface through the detection of nitrogen, which is a key element in the molecular structure of these compounds. The higher carbon content and lower oxygen content on the inhibited surfaces compared to the corroded surface in HCl further support the formation of a protective organic film that prevents oxidation of the metal.

#### 3.9.2. FTIR Spectroscopy

**Figure 15:**
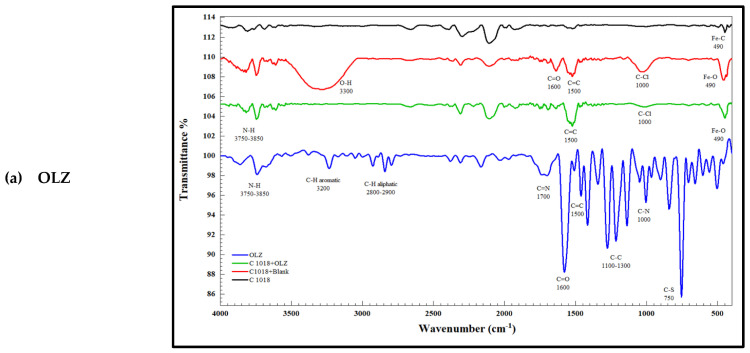

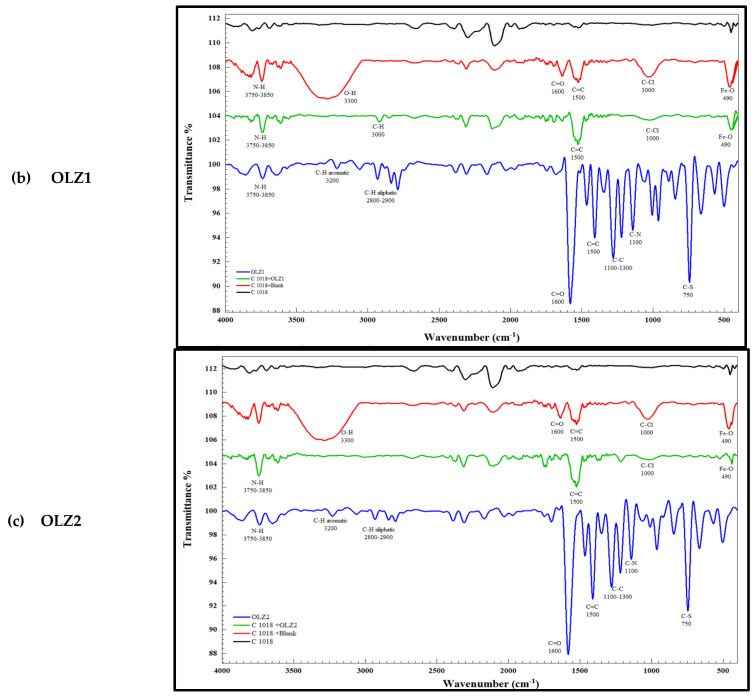
FTIR spectra of C1018 and corrosion products after 6 h of immersion with and without an inhibitor: (**a**) OLZ, (**b**) OLZ1, and (**c**) OLZ2.

FTIR spectroscopy was employed to characterize the molecular structure of the inhibitors and to identify potential functional groups involved in the adsorption process on the metal surface. The results of FTIR analysis of pure OLZ, OLZ1, and OLZ2 are presented in Figure 15. The spectra reveal characteristic absorption bands corresponding to various functional groups present in the inhibitor molecules. All three compounds exhibit bands in the region of 3200–3500 cm^−1^ attributed to N-H^−^ stretching vibrations, bands around 2900–3000 cm^−1^ due to C-H stretching, and bands in the region^−^ of 1600–1700 cm^−1^ assigned to C=O stretching vibrations. The spectra of OLZ1 and OLZ2 show^−^ additional bands compared to the parent OLZ, confirming the successful incorporation of acrylamide and ethyl acetate groups, respectively. The blank solution showed a peak at 3300 cm^−1^ that was due to O-H stretching, but it was absent in the presence of the inhibitors. The -OH, -C=N, C=C, and aromatic ring on the surface of the metal serve as active sites for the interaction of the inhibitors.

### 3.10. Inhibition Mechanism

Olanzapine and its derivatives represent a promising pharmaceutical compound in the field of corrosion inhibition, combining high efficacy with environmental sustainability. The molecular structure of olanzapine features multiple heterocyclic systems containing nitrogen and sulfur atoms, along with delocalized π-electron systems, providing multiple adsorption sites on metallic surfaces. These combined features are hardly common in most organic molecules used as corrosion inhibitors.

The analysis of corrosion inhibition depends on how inhibitors adsorb onto steel surfaces. Organic compounds adsorb to surfaces through physical or chemical methods, but both mechanisms can happen at the same time. The adsorption process depends on three main factors, which include inhibitor charge, molecular structure, and metal surface properties. The inhibitor adsorption process relies on functional groups such as aromatic rings (N-H, C=O, C=C, and C-O) and N present in OLZ and its derivatives, which act as adsorption centers. The electron transfer capability of these functional groups to metal d-orbitals plays a crucial role in the adsorption process [66].

FTIR spectral data delivers essential information about how the studied compounds inhibit the corrosion process, as shown in Figure 15 and Table 8. It is clear in Figure 15 that the inhibited samples show diminished O-H stretching bands at 3300 cm^−1^, which indicates that inhibitor molecules move water molecules off the metal surface to block their involvement in corrosion reactions. This displacement is a crucial step in the inhibition process, as water molecules are essential for the electrochemical reactions that drive corrosion.

The decreased intensity of Fe-O peaks at 490 cm^−1^ in the presence of inhibitors proves that inhibitors block the formation of iron oxides, which act as primary corrosion products. Inhibitor molecules adsorb on metal surface active sites to block both anodic and cathodic reactions that occur during corrosion. By impeding these electrochemical reactions, the inhibitors effectively reduce the corrosion rate.

FTIR spectra of corrosion products show that inhibitor-specific peaks persist, which demonstrates that inhibitors maintain their adsorption to metal surfaces throughout the testing duration. Multiple adsorption mechanisms operate based on the various functional groups found in inhibitor molecules [67,68]. The proposed mechanism in OLZ is shown in Figure 15, where the immersion of steel coupons in the blank HCl resulted in metal dissolution, whereas the addition of OLZ resulted in inhibitor adsorption on the steel surface through chemisorption and physisorption. The adsorption process receives additional support from electrostatic forces between charged inhibitor molecules and metal surfaces, especially in acidic conditions where the metal surface develops a positive charge and inhibitor molecules become protonated. This type of interaction corresponds to physisorption and is generally weaker than chemisorption but can still contribute significantly to the overall inhibition effect. The spectra of OLZ1 and OLZ2 show additional bands compared to the parent OLZ, confirming the successful incorporation of acrylamide and ethyl acetate groups, respectively.

## 4. Conclusions

This study determined the inhibition of corrosion for carbon steel in 1 M HCl solution using a comprehensive suite of electrochemical and surface analytical techniques by olanzapine (OLZ) and two novel derivatives, OLZ1 and OLZ2. This study determined the following results from the experimental data obtained:

1. The three inhibition properties of OLZ and its derivatives on C1018 carbon steel in 1 M HCl solution were very high at 300 ppm, where inhibition efficiencies exceeded 88%. However, inhibition efficiency was temperature- and concentration-dependent.
2. Electrochemical studies indicate that the three inhibitors are predominantly anodic. PDP-derived inhibition efficiencies agreed with those obtained from EIS measurements.
3. At 298 K, the inhibition efficiency followed the order OLZ2 > OLZ1 > OLZ, while at 318 K, it followed the order OLZ1 > OLZ2 > OLZ, which confirms that the structural modifications enhanced the corrosion inhibition properties of the parent molecule.
4. The SEM-EDX analysis demonstrates that OLZ and its derivatives effectively inhibit steel corrosion in 1 M HCl compared to the uninhibited solution.
5. The FTIR spectroscopy results show that the metal surface active sites include -NH, -C=N, C=C, and aromatic ring structures.

## Figures and Tables

**Table 2:** Weight loss data of carbon steel showing the corrosion rate and inhibition efficiency in the absence and presence of the inhibitors in 1 M HCl at 298 K and 318 K.

Inhibitor	At 298 K				At 318 K			
	△ w	C.R		IE%	△ w	C.R		IE%
		mpy	mmpy			mpy	mmpy	
Blank	1.034	1876.08	47.63	-	2.875	5361.45	136.13	-
OLZ	0.07	131.7	3.34	92.98%	0.688	1353.4	34.36	74.75%
OLZ1	0.092	172.05	4.36	90.82%	0.495	984.8	25	81.63%
OLZ2	0.035	70.51	1.79	96.23%	0.541	1101.85	27.97	79.44%

**Table 3:** The data obtained from fitted EIS curves for corrosion control of carbon steel with the presence and absence of OLZ, OLZ1, and OLZ2 at 300 ppm.

Inh.	R_s_ Ω·cm^2^	CPE_dl_		R_ct_ Ω·cm^2^	CPE_f_		R_f_ Ω·cm^2^	C_dl_ μF·cm^−2^	R_p_ Ω·cm^2^	IE %	STD IE	χ^2^ × 10^−3^
		Y_01_ (mΩ s^n^ cm^−2^)	n_1_		Y_02_ (mΩ s^n^ cm^−2^)	n_2_						
Blank	131.15	89.380	0.867	1.74	70.465	0.958	8.005	193.850	140.897	-	-	9.94
OLZ	0.74	63.657	0.967	10.89	92.736	0.882	1189.333	79.578	1200.97	88.28	0.0940	4.14
OLZ1	1.64	47.53	0.972	1416.3	20.820	0.984	12.449	65.471	1430.42	90.15	0.1956	3.21
OLZ2	1.32	50.41	0.996	898.06	29.413	0.904	747.033	51.416	1646.42	91.45	0.2043	5.14

**Table 4:** Results for the inhibitory effect of OLZ, OLZ1, and OLZ2 on carbon steel by potentiodynamic polarization and linear polarization resistance experiments.

Inhibitor	PDP						LPR		
	E_corr_ (mV/SCE)	i_corr_ (μA cm^−2^)	β_a_ (mV/dec)	β_c_ (mV/dec)	IE %	χ^2^ × 10^−3^	R_p_ Ω·cm^2^	IE %	STD CR
Blank	−447.00	200.00	160.80	120.00	-	23.93	132.20	-	-
OLZ	−401.000	20.100	100.00	132.700	89.95	191.6	1135.66	88.35	0.0655
OLZ1	−397.000	15.900	89.000	218.600	92.05	75.34	1473.00	91.01	0.2311
OLZ2	−422.000	14.600	91.400	241.600	92.70	821.1	1760.66	92.47	0.3597

**Table 5:** Quantum chemical indices of the molecules.

Parameters	Inhibitor Molecules		
	OLZ	OLZ1	OLZ2
E_HOMO_ (eV)	−4.643	−4.637	−4.669
E_LUMO_ (eV)	−1.865	−1.852	−1.879
I (eV)	4.643	4.637	4.669
A (eV)	1.865	1.852	1.879
ΔE (eV)	2.778	2.785	2.790
χ (eV)	3.254	3.245	3.274
η (eV)	1.389	1.393	1.395
ΔN (eV)	−2.697	−2.697	−2.671
μ (Debye)	4.603	6.510	5.160

**Table 6:** Diffusion coefficients of the corrosive particles in films.

Inhibitor Film	D (×10^−9^ m^2^·s^−1^)		
	H_2_O	H_3_O^+^	Cl^−^
Blank	2.79	2.57	1.24
OLZ	0.39	0.35	0.07
OLZ1	0.66	0.87	0.25
OLZ2	0.35	0.17	0.19

**Table 7:** Atomic percentage of metal in the presence and absence of OLZ, OLZ1, and OLZ2 by EDX.

Element	Metal	Metal with HCl	Metal with OLZ	Metal with OLZ1	Metal with OLZ2
N	1.57	0.91	1.74	1.68	1.68
O	1.69	20.76	1.62	1.78	2.24
C	9.69	14.42	11.41	10.75	10.81
S	0.46	0.36	0.43	0.43	0.39
P	0.65	0.51	0.59	0.73	0.67
Mn	0.38	0.33	0.31	0.45	0.40
Fe	85.56	62.71	83.90	84.19	85.49

**Table 8:** FTIR band assignments for corrosion products and inhibitor effects.

NO.	Band	Absorbance Peak	Reason
1	Fe-O	450	The presence of a sharp, intense peak indicates the formation of ferrous and ferric oxides or hydroxides
2	O-H	3300	The presence of a hydroxyl group is an indication of the formation of ferric oxides or hydroxides
3	C-Cl	1000	Presence of chloride ions as corrosion products
4	Fe-O	490	Reduction in peak intensity due to the presence of the inhibitor
5	C-Cl	1000	Reduction in peak intensity due to the presence of the inhibitor
6	O-H	3300	Disappearance of the hydroxyl group in the presence of the inhibitor

## Data Availability

The data presented in this study are not publicly available due to privacy restrictions related to the personal information of the participants. However, data can be made available upon reasonable request from the corresponding author.

## Supplementary Materials

The following supporting information can be downloaded at https://www.mdpi.com/article/10.3390/ma18122902/s1. Figure S1: Fourier Transformer Infrared Spectra of OLZ, OLZ1, and OLZ2. Figure S2: Nuclear Magnetic Resonance Spectra of (**a**) OLZ, (**b**) OLZ1, (**c**) OLZ2. Table S1: Chemical shifts of 1H NMR spectra of compounds OLZ, OLZ1, and OLZ2.
