# Supplementary material for: Towards a Sustainable Material Protection: Olanzapine Drugs and Their Derivatives as Corrosion Inhibitors for C1018 Steel in 1 M Hydrochloric Acid
Source: Materials (Basel). 2025 Jun 19;18(12):2902. doi: 10.3390/ma18122902 (PMC12195516; doi:10.3390/ma18122902)
Supplement: Supplementary file 1 [file materials-18-02902-s001.zip › materials-3654856-supplementary.pdf]

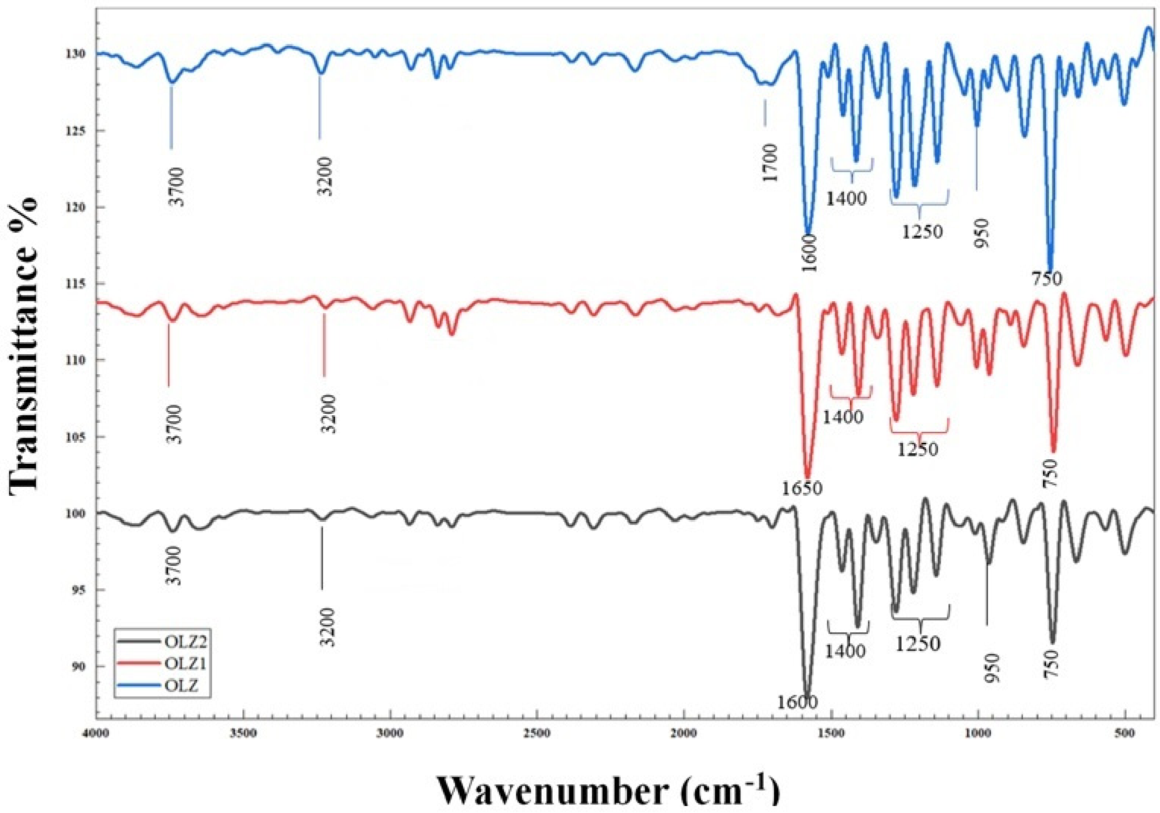

Figure S1: Fourier Transformer Infrared Spectra of OLZ, OLZ1, and OLZ2.

Table S1: Chemical shifts of 1H NMR spectra of compounds OLZ, OLZ1, and OLZ2.

Chemical Structure	Peak	Relative Proton	Chemical Shift (ppm)
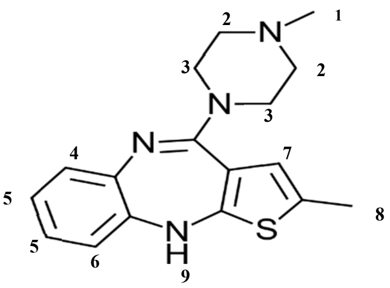	1	N-CH_3	1.4
	2	N-CH_2	2.25
	3	N-CH_2	2.18
	4	C-H (benzene ring)	7.1
	5	C-H (benzene ring)	7.00
	6	C-H (benzene ring)	6.6
	7	C-H (thiophene ring)	6.5
	8	-CH_3 (thiophene ring)	3.46
	9	N-H	5.04
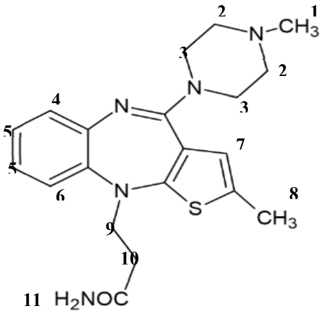	1	N-CH_3	1.4
	2	N-CH_2	2.4
	3	N-CH_2	2.2
	4	C-H (benzene ring)	7.1
	5	C-H (benzene ring)	7.0
	6	C-H (benzene ring)	6.9
	7	C-H (thiophene ring)	6.5
	8	-CH_3 (thiophene ring)	1.7
	9	N-CH_2	2.6
	10	C-CH_2	3.6
	11	NH_2 (amide group)	5
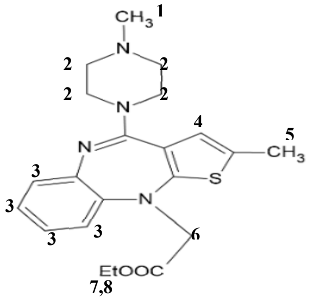	1	N-CH_3	1.4
	2	-N-CH_2	2.3-2.8
	3	C-H (benzene ring)	7
	4	-CH (thiophene ring)	6.8
	5	CH_3 (thiophene ring)	3.5
	6	N-CH_2	6.3
	7	-CH_2- (ethanoate group)	5.5
	8	-CH_3 (ethanoate group)	2.3

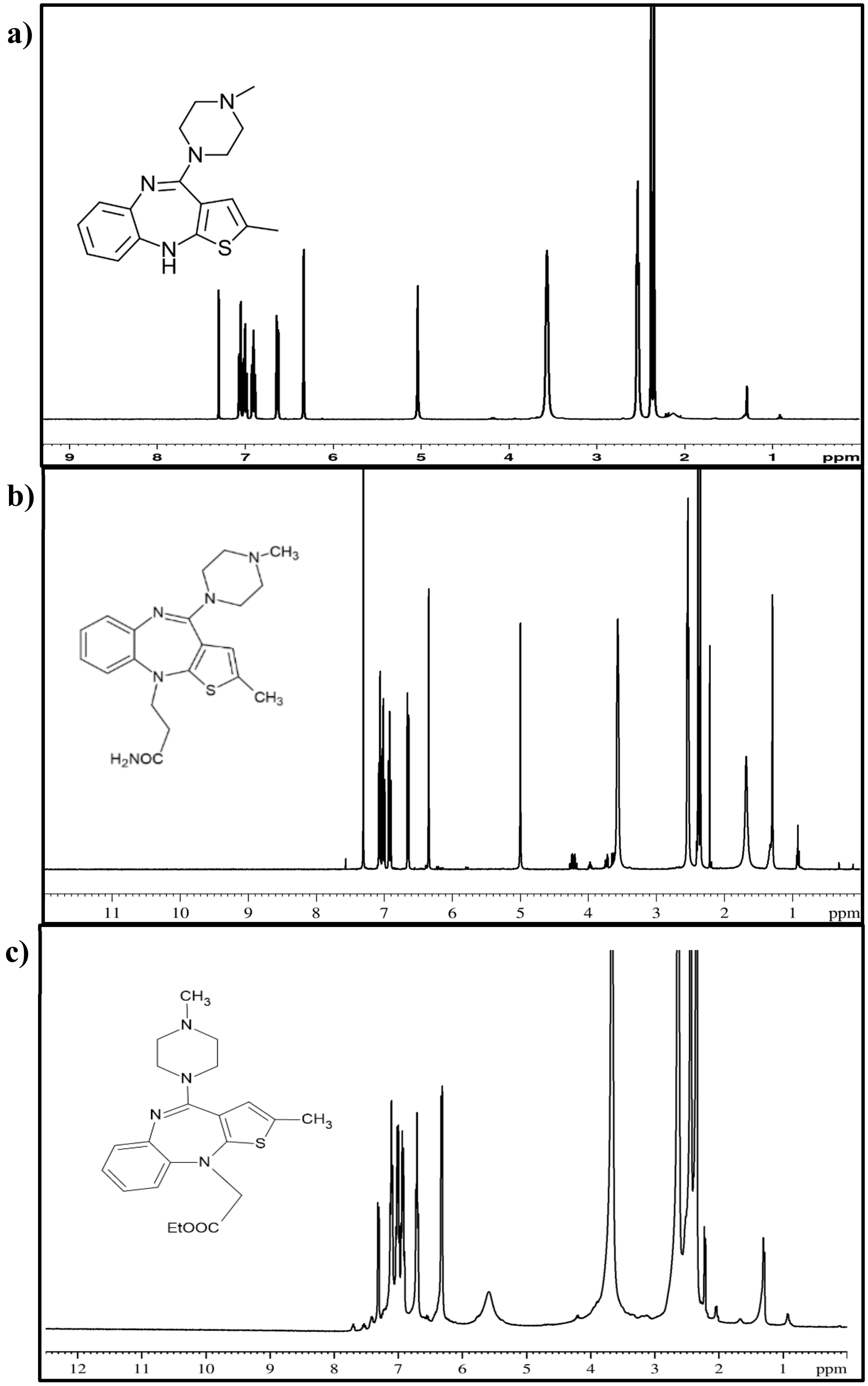

Figure S2: Nuclear Magnetic Resonance Spectra of a) OLZ, b) OLZ1, c) OLZ2.
